# Let’s not get ahead of ourselves: we have no idea if moral reasoning causes moral progress

**DOI:** 10.1080/13869795.2024.2363876

**Published:** 2024-06-26

**Authors:** Paul Rehren, Charlie Blunden

**Affiliations:** Department of Philosophy and Religious Studies, Ethics Institute, Utrecht University, Utrecht, The Netherlands

**Keywords:** moral reasoning, moral progress, moral psychology, causal inference, empirically-informed philosophy

## Abstract

An important question about moral progress is what causes it. One of the most popular proposed mechanisms is moral reasoning: moral progress often happens because lots of people reason their way to improved moral beliefs. Authors who defend moral reasoning as a cause of moral progress have relied on two broad lines of argument: the general and the specific line. The general line presents evidence that moral reasoning is in general a powerful mechanism of moral belief change, while the specific line tries to establish that moral reasoning can explain specific historical examples of moral progress. In this paper, we examine these lines in detail, using Kumar and Campbell’s (2022, *A Better Ape: The Evolution of the Moral Mind and How It Made Us Human*. Oxford University Press) model of rational moral progress to sharpen our focus. For each line, we explain the empirical assumptions it makes; we then argue that the available evidence supports none of these assumptions. We conclude that at this point, we have no idea if moral reasoning causes moral progress.

What causes moral progress? Competing proposals abound. Moral progress might be driven by people’s moral psychology plastically adapting to safer and more disease-free environments (Buchanan & Powell, 2018), it might be driven by new knowledge about social possibilities gained from experiments in living (e.g. Anderson, 2014) or by social struggles which react to structural contractions and crises (Jaeggi, 2021), for example.

One mechanism that's very popular among philosophers and social scientists alike is moral reasoning (e.g. Bloom, 2010, 490; Killen & Dahl, 2021, 1216–1217; Kumar & Campbell, 2022, 13; Pinker, 2011, 657–658; Singer, 2011, 119). The central idea of these proposals is that progress often happens because people’s moral beliefs^1^ improve through moral reasoning, deliberation or reflection, where this could involve adopting a new belief (e.g. that slavery is wrong), rejecting an old belief (e.g. that slavery is just) or a change in the level of confidence in an existing belief.

We need to draw an important distinction here: Moral reasoning can function both as a *direct* and an *indirect* cause of moral progress. Moral reasoning is a direct cause of moral progress if it causes enough individuals to change their moral beliefs so that these belief changes together add up to society-level moral progress. In contrast, reasoning is an indirect cause of moral progress if it in itself only leads a small number of people to change their moral beliefs, but these progressive moral belief changes then go on to spread throughout society at large through other mechanisms – for example, prestige bias, economic or legal pressures, rhetoric, or social conformity.

Most authors think of moral reasoning as a direct cause of moral progress. Kumar and Campbell (2022), for example, explicitly exclude from their model indirect mechanisms like ‘random cultural drift or […] the influence of prestigious figures’ (196). Likewise, Pinker argues that many past moral improvements were ‘powered not just by the sporadic appearance of outstanding thinkers but by a rise in the quality of everyone’s thinking’ (2011, 650; also, see, Killen & Dahl, 2021, 1210; Bloom, 2010, 490). This, then, shall be the focus of this paper: accounts of moral reasoning as a direct cause of moral progress.

There are three main motivations for focusing on moral reasoning as a direct cause of moral progress. First, moral reasoning has long been seen as a central shaper of human moral cognition, and so it makes sense to view it as a potential candidate cause of large-scale moral belief change. Second, some have argued that we should prefer moral reasoning to other potential causes of moral progress. According to Kumar and Campbell, for example, rational moral progress will be more durable than other forms of moral progress (e.g. progress that result from random cultural drift or the influence of prestigious figures) because it is based on an appreciation of good reasons for one’s moral beliefs (2022, 196). Third, unlike many other candidate causes of moral progress, moral reasoning can be targeted relatively easily by interventions – an attractive feature for moral progress theorists who would like their work to make a real world difference.

While we get the appeal, it's an empirical question if and to what extent moral reasoning is indeed a direct cause of moral progress. In the literature, we find two main lines of argument. The *general line* aims to provide evidence that moral reasoning is in general a powerful mechanism of moral belief change; this, so goes the argument, makes it (at least) a plausible candidate mechanism for moral progress (e.g. Killen & Dahl, 2021, 1216–1217; Pinker, 2011, 642–670; Singer, 2011, 191–198). The *specific line* aims to show that moral reasoning was a cause of specific, historical episodes of progressive moral change (e.g. Bloom, 2010; Pinker, 2011, 642–670; Tam, 2020, 94–96).

In this paper, we describe both lines and explain the empirical assumptions they make (sometimes explicitly, sometimes implicitly). We then argue that the available evidence fails to establish any of these premises. As our main example, we use Kumar and Campbell’s (2022) recent theory of moral progress. We focus on this theory for two reasons. First, among moral reasoning theories, Kumar and Campbell’s model is the most extensively developed and defended in the literature to date. These authors have published on consistency reasoning and its progressive potential for more than a decade (Campbell, 2014; Campbell & Kumar, 2012; 2013; Kumar & Campbell, 2022). As such, they have fleshed out their account to a greater extent than other moral reasoning accounts in the literature, which, in our view, justifies focussing on their account as the prime example of an empirically-informed moral reasoning theory. Second, Kumar and Campbell use both the general and the specific lines in defending their theory. This makes their work an instructive case study of the promise and pitfalls of these different lines of argument. Nonetheless, what we have to say is relevant for many other models of moral progress that invoke moral reasoning; we will make this clear throughout the paper.

## 1. The model of rational moral progress

Moral progress happens when things^2^ improve morally over time (Jamieson, 2002, 318). There are various different types of moral progress (for an overview, see, Sauer, Blunden, Eriksen, & Rehren, 2021). Kumar and Campbell, along with most of the authors whose work we discuss in this paper, focus on two types. First, *inclusive moral progress*, which ‘occurs when morality encompasses people who were once regarded as outsiders, reducing moral exclusivity between groups’ (185; see, also, Bloom, 2010; Killen & Dahl, 2021, 1216–1217; Tam, 2020, 94–96). Examples of this include the elimination of chattel slavery and the African slave trade, and the reduction in prejudice and discrimination on the basis of race and ethnicity. Second, *egalitarian moral progress*, which ‘reduces domination/subordination and advances moral equality’ (187; also, see, Kitcher, 2021, 13; Bloom, 2010; Killen & Dahl, 2021, 1216–1217). Examples include the wider recognition that women have interests equal in value to those of men, and the greater acceptance of gay people.

How does moral reasoning cause moral progress? In a nutshell, the idea is that moral reasoning has progressive potential because it allows people to identify and resolve moral inconsistencies. Killen and Dahl (2021, 1210), for example, argue that moral reasoning facilitates ‘people noticing inconsistencies in principles or unequal treatment of others.’ Tam (2020) agrees: ‘[t]he articulation of abstract principles and rational arguments helps to reveal inconsistencies and falsehoods in the status quo’ (also, see, Singer, 2011, 118–119; Kitcher, 2021, 13).

Inconsistency is also central to Kumar and Campbell’s model of moral progress. At the core of that model is a type of moral reasoning called consistency reasoning (CR): ‘consistency reasoning […] isn’t just of historical interest. Positive feedback loops between reasoning and social institutions not only fostered morally evolved societies in the recent past but promise to do so in the future as well’ (2022, 13). As input, CR takes sets of particular moral cases that ‘are, by one’s own lights, similar in morally relevant respects’ (Campbell & Kumar, 2012, 274). Since people tend to dislike moral inconsistency and strive to ‘treat like cases alike’ (296), when it becomes clear to them that their own moral beliefs are inconsistent, they face a choice: ‘either accept inconsistency and thereby give up any semblance of having a good reason for either of the responses or else revise the less tenable response’ (284). CR produces moral belief changes when people go with the second option and revise one of their moral beliefs.

Not all moral belief changes are progressive, however. Kumar and Campbell recognize this, but argue that CR will cause moral belief improvement under the right conditions: ‘when people form accurate beliefs about the world around them and those who inhabit it, they tend to re-evaluate their moral feelings and norms in ways that lead them rationally toward greater inclusivity and equality’ (2022, 195–196). For progress to happen, then, people need to first arrive at more accurate factual beliefs about the ‘the world around them and those who inhabit it.’ What factual beliefs are these? The examples Kumar and Campbell provide fall into two broad categories (2022, 205–220). In the first category are beliefs about the characteristics of individuals or groups that bear on whether there are morally relevant differences between them and other individuals or groups (e.g. women are able to excel in high status roles; not all poor people are lazy). In the second category are beliefs about the way other people or groups are being treated – in particular, about the harms they suffer (e.g. anti-black racism causes many black people great harm; animals in factory farms are treated horribly).

According to Kumar and Campbell, when people’s grasp of these and similar facts improves, this can then lead them to consistency reason their way to greater inclusivity and equality: ‘when people are collectively well informed,’ rational progressive moral change is possible

when their [i.e. people’s] social and institutional environments allow them to reason fruitfully together […]. Under these conditions, people can wisely re-interpret their moral norms and appropriately resolve conflicts between them. Rational influences on the moral mind can thereby tilt societies toward moral progress. (2022, 196)

In short: CR leads to moral progress when the right factual beliefs change in the right way and the circumstances encourage people to engage in CR.

### 1.1. How much progress is supposed to be explained?

Before we begin, it is worth asking about the scope of Kumar and Campbell’s model, and of other models like it. In the literature, moral progress is almost always understood to be a feature of populations (often large populations) rather than individuals. Few would say, for example, that a single person who used to believe that factory farming is unproblematic but now believes it’s a horrible practice, is an instance of moral progress. Instead, moral progress requires group-level moral belief change: it is not enough for one or two people to change their moral beliefs for the better, but a substantial proportion of the members of the group in question needs to do so.^3^ Kumar and Campbell themselves grant that there are many other plausible causes of group-level progressive belief change that their model does not capture. For instance, when arguing that rational moral change played a large role in expanding gender equality, Kumar and Campbell note that they ‘are not offering a thorough explanation of moral progress’ and that ‘[a] wider range of cultural, political, and legal forces have fostered, and constrained, the evolution of gender equality’ (2022, 233). Other authors who argue for moral reasoning as an important direct cause of moral progress caveat their views in a similar way (e.g. Bloom, 2010, 490; Pinker, 2011, 573).

While we are happy to see these caveats, they do raise an important question: how much moral progress is moral reasoning supposed to explain? Or, to put it a little more quantitatively: What proportion of progressive moral belief changes is moral reasoning supposed to have caused? To illustrate, take the sharp decline in homophobic attitudes in North America and Western Europe over the last five decades or so (an often cited historical example of moral progress). Say it turned out that moral reasoning was the main cause of 10% of these attitude changes. Is that enough for moral reasoning to count as an important, direct cause of progress? Maybe. But what about 1%? Half a percent? Maybe not. It strikes us, then, that Kumar, Campbell and other authors with similar views at least need to claim that moral reasoning causes a non-negligible proportion of moral belief improvements, in sense that if no one engages in moral reasoning, this would noticeably slow down or even stall the course of moral change.

Our main reason for bringing up these questions is that depending on the answers, the burden of the empirical evidence needed to establish moral reasoning as a plausible direct cause of moral progress changes considerably. For example, below, we will argue that for the general line to be convincing, one piece of evidence we’d need to see is that in the real world, enough people engage in moral reasoning often enough. Clearly, what ‘enough’ means in this sentence has a lot to do with the proportion of progressive moral belief changes one’s model of moral progress is supposed to account for. While we will not press this point further, we think it may be useful to keep it at the back of your mind for the discussion that follows.

## 2. The general line

Kumar and Campbell argue that CR is a powerful mechanism of moral belief change, in general: ‘[t]here is plenty of evidence that people actually change their moral opinions in response to consistency reasoning’ (2022, 119). This makes CR a plausible candidate-cause of progressive moral belief changes, as well (for other examples, see, Singer, 2011, 191–198; Killen & Dahl, 2021, 1216–1217; Pinker, 2011, 642–670). Recall that we call this line of evidence the general line.

### 2.1. Kumar and Campbell’s evidence

As far as we can see, across all of their publications on the topic, Kumar and Campbell cite a total of three papers (Petrinovich & O’Neill, 1996; Schwitzgebel & Cushman, 2012; 2015) to show that ‘people actually change their moral opinions in response to consistency reasoning’ (2022, 119). Each of these papers investigated the influence of order effects on people’s moral judgments. Order framing effects involve two or more scenarios. In all of the scenarios, optional actions have the same outcomes (e.g. 1 versus 5 deaths), but produce those outcomes in different ways. Participants then read and make moral judgments about these scenarios one after the other; what is manipulated between the conditions is the order in which this sequence of scenarios is presented. An order effect is present if participants make (or would make) different moral judgments about the same scenario depending on the order in which the sequence of scenarios was presented.

The order effects that Kumar and Campbell point to all involve versions of the well-known trolley dilemma. To illustrate, let us look at Schwitzgebel and Cushman (2012). Their study used two versions of the trolley dilemma. Both versions asked participants to make a moral judgment about an action that would save five lives at the expense of one (participants rated the action on a seven-point scale, from ‘extremely morally good’ to ‘extremely morally bad’). In Push-type scenarios, the action involved ‘killing one person through direct physical contact as a means of saving five people.’ In Switch-type scenarios, the action instead involved ‘one person’s dying, without direct physical contact from the agent, as a side effect of an action to save five’ (both, 2012, 138). Participants read and rated two pairs of these scenarios (one Switch-type, one Push-type), in random order. Schwitzgebel and Cushman found that presentation order had a significant effect on participants’ moral judgments: ‘respondents were more likely to rate the Push and Switch scenarios equivalently when Push was presented before Switch’ (141). The other two articles Kumar and Campbell cite report similar results, as do a number of other studies (see, Rehren & Sinnott-Armstrong, 2021, 612–613).

According to Kumar and Campbell, these findings provide evidence that CR is an important cause of moral belief change. This is because they suggest that order effects reveal the influence of CR: ‘A natural explanation for this result is that subjects perceive that there are no morally relevant differences between the two cases and infer that whatever one should do in the one case, one should also do in the other’ (Campbell & Kumar, 2012, 285–286).

### 2.2. Is the evidence even relevant?

On its face, none of the studies Kumar and Campbell cite investigate the influence of CR on moral belief change – none of these studies even mention CR. Kumar and Campbell acknowledge this, but still think that findings of order effects support their model because CR provides a ‘natural explanation’ (Campbell & Kumar, 2012, 285) for these effects. So, does it?

We doubt it. One major problem with Kumar and Campbell’s claim is that there are already a number of plausible competing explanations for order effects out there in the literature (for a brief overview, see, Wiegmann & Waldmann, 2014, 39–40). For example, Horne, Powell, and Spino (2013) present evidence that order effects involving a Switch-type and a Push-type scenario are the result of asymmetric belief updating in light of new evidence. Others have explained order effects involving trolley dilemmas as transfer effects triggered by the transfer of salient causal structure between dilemmas (Wiegmann & Waldmann, 2014). Contrast this with Kumar and Campbell’s explanation, which they provide no empirical evidence for. We are, then, not convinced that the evidence Kumar and Campbell provide is about what it would need to be about to support their model of moral progress.

Even if we grant that the research Kumar and Campbell cite can tell us something about the role of CR in moral cognition, another issue remains. The two main types of moral progress Kumar and Campbell are interested in have to do with increasing equality and inclusivity (Kumar & Campbell, 2022, 185–187). However, these are not the domains of morality investigated in the studies they cite. Instead, these studies all rely on trolley dilemmas. Many moral psychologists have cautioned that generalizing findings with trolley dilemmas to other domains of morality and to every day moral recognition is deeply fraught (e.g. Bauman, McGraw, Bartels, & Caleb, 2014; Bostyn, Sevenhant, & Roets, 2018; Hester & Gray, 2020).

Kumar and Campbell are not the only moral progress theorists who rely on evidence of questionable relevance. For example, to support the claim that ‘moral reasoning enables youths and adults to challenge unfair societal arrangements,’ Killen and Dahl (2021) cite evidence that ‘adolescents view extreme levels of social stratification as a product of a society that's not equal and a government that will favor some groups over other groups’ (both, 1216). It is hard to see what this result has to do with moral reasoning, and Killen and Dahl do not explain. Conversely, Pinker (2011, 650–656) cites research on the Flynn effect – the finding that during the twentieth century, there has been a marked and steady increase in intelligence test scores measured in many parts of the world. Yet it is not obvious that intelligence is closely related to the frequency with which people engage in moral reasoning or its power to change their moral beliefs (see, Bostyn, De Keersmaecker, Van Assche, & Roets, 2020; Sauer, 2018).

The issue of external validity likewise is a concern for other reasoning-based explanations of moral progress. Like Kumar and Campbell, these accounts tend to focus on gains to inclusivity, equality or both. At the same time, research on moral reasoning (like research on order effects) has relied heavily on trolley dilemmas. For example, a recent systematic review on the effects of domain-general reasoning on moral judgments found that almost 80% of published studies had used sacrificial dilemmas (Rehren, 2022; also, see, Patil et al., 2021; Paxton & Greene, 2010). Again, if the results of studies with sacrificial dilemmas do not straight-forwardly generalise to moral domains like inclusivity and equality, then moral reasoning theories of moral progress should not rely on these results.

### 2.3. A direct cause of moral progress

Let’s assume that CR explains 100% of moral order effects. In that case, should the evidence Kumar and Campbell present convince us of their model of rational moral progress? We don’t think so. Moral progress is group-level moral belief change for the better. Therefore, the research doesn’t only need to be informative about the effect of CR on moral beliefs; instead, it needs to support the claim that CR is a plausible candidate for a direct cause of group-level moral belief change.

One consideration that is relevant here is effect size. The kinds of moral belief changes Kumar and Campbell (and many other moral progress theorists) try to explain are quite drastic – people used to believe *p* (e.g. slavery is just; gay couples should not be allowed to marry; black people are inferior to white people), but came to believe *not-p* (e.g. slavery is unjust; gay couples should be allowed to marry; black people are not inferior to white people). Therefore, it would be most convincing if psychological research on moral order effects revealed that presentation order can often produce moral belief changes of something like this magnitude.

In fact, however, the effects that Kumar and Campbell point to are rather small. To illustrate, recall that Schwitzgebel and Cushman (2012) used a seven-point scale to measure their participant’s more judgements. They found that when participants read the Push-type dilemma first, this increased their rating of the Switch-type dilemma by half a point on average compared to participants who read the Switch-type dilemma first. This difference amounts to less than 10% of the rating scale these researchers used – not very impressive if we’re trying to explain substantial moral belief change. Matters are similar for the other experiments Kumar and Campbell cite.

These issues are not limited to the evidence for CR. Moral psychologists have used two main approaches to study the effect of (domain-general) reasoning on moral beliefs. The first approach investigates if individual differences in people’s tendency to engage in reflective (or intuitive) processing predict differences in their moral belief patterns (for an overview, see, Patil et al., 2021). Most research that uses this approach focuses on whether reasoning leads to more utilitarian (compared to deontological) moral beliefs; overall, the evidence is mixed: ‘Many studies do find a positive association between reasoning measures and utilitarian tendencies […], but others do not […], and some provide mixed or inconsistent findings’ (Patil et al., 2021, 445).

The second approach uses experimental manipulations designed to either encourage participants to engage in reflection, or to inhibit their ability to reflect and therefore make them fall back on a more intuitive mode of processing. A recent systematic review (Rehren, 2022) of the literature found that ‘[m]ost of the studies included in the review did not find a significant effect on moral judgment of experimentally encouraging or inhibiting reflective processing’ (38). Moreover, a meta-analysis of the largest subset of these studies did not find evidence that reflection leads people to judge the consequentialist option in sacrificial moral dilemmas more favourably (after correcting for publication bias). Reasoning’s power to bring about drastic moral belief changes, then, looks to be limited, more generally.

This does not, however, rule out moral reasoning (including consistency reasoning) as a potential direct cause of moral progress. One way this could be true is if moral belief changes brought about by moral reasoning tend to stack. Say someone starts out with a moral belief. Engaging in reasoning once is not likely to change their mind; however, it may reduce their confidence in this moral belief and so push them a little towards giving it up. If this change persists and the person then reasons about the issue again, this may then further weaken their confidence, and so on. In this way, even if the effect of reasoning on moral beliefs that we see in psychological studies is only small, over time, reasoning may still cause substantial moral belief revision.

This is a possible story, but one that does involve a number of empirical assumptions about the way moral reasoning works. One is that people engage in moral reasoning with some regularity; otherwise, there won't be enough opportunities for substantial stacking to happen. A second assumption is that when reasoning causes moral belief changes, these changes tend to persist – again, without this, it is hard to see how small reasoning-induced moral belief changes should be able to stack.

Are these assumptions reasonable? Unfortunately, we have no idea. Regarding consistency reasoning, clearly, the studies that Campbell and Kumar cite cannot help us estimate how often people engage in CR in everyday life, or how often these episodes of reasoning have an effect on their moral beliefs. Likewise, to get a systematic idea of if (and to what extent) moral belief changes that come about from CR persist, we would require time-series data – that is, multiple observations at different points in time of how CR impacts the moral beliefs of the same individuals. Yet as far as we are aware, no research like this exists. At present, then, it would be pure conjecture to invoke stacked incremental gains to argue for CR as a plausible direct cause of moral progress.

The situation is not much different for the literature on moral reasoning more broadly. While the question of how often people engage in moral reasoning in the real world has been a matter of some debate, there is little convincing evidence either way. To figure out how often and with what outcomes people engage in moral reasoning in everyday life, experiments in the lab simply will not do. Instead, researchers need to take ‘the study of morality out of the lab and into the stream of life’ (Graham, 2014, 1242). We are not aware of any studies like this that focus on moral reasoning.

Likewise, we know of no research that would be able to tell us if moral belief changes caused by moral reasoning typically persist over time. While it certainly seems natural to expect that when moral beliefs change in light of reasoning, these changes will last, to show this systematically, we would again need time series data – as far as we are aware, no studies of this sort currently exist.

## 3. The specific line

In the previous section, we have argued that Kumar and Campbell’s general line of support for their model of moral progress is unconvincing. We also showed that other attempts to establish moral reasoning as a direct cause of moral progress face many of the same hurdles.

In this section, we will turn to the specific line, which aims to show that moral reasoning was a direct cause of specific historical cases of moral progress. The case studies Kumar and Campbell describe include the legal abolition of chattel slavery, the decline of exclusionary anti-black racism, the decline of homophobia, the ongoing social movements against speciesism, and increases in gender equality. In these historical case studies, the beliefs, attitudes, norms and so on of many people really did change in progressive ways. Kumar and Campbell argue that their model can provide a causal explanation for these changes (see, 2022, 195–196): in these historical periods, it was CR that caused many people to change their minds.

Kumar and Campbell’s case for this claim involves two steps (also, see, Killen & Dahl, 2021, 1217; Kitcher, 2021). The first step is to establish a causal relationship between a predictor other than moral reasoning – improved factual beliefs about the world and the people in it – and more progressive moral beliefs. The second step is to show that CR can explain this relationship: when people’s grasp of the relevant factual beliefs improves, many of them then engage in CR which has the effect of changing their moral beliefs in a progressive direction.

Other authors have argued for their reasoning-based accounts of moral progress in a similar way, though in the first step, they focus on establishing a different causal relationship: one between public speech (broadly understood) and progressive moral belief changes. For example, Tam (2020) argues that ‘British abolitionists were successful because they gave compelling We-reasons to Britons to redefine the social norm of British honor’ (94). Along similar lines, Killen and Dahl (2021), to explain recent ‘protests and resistance about climate change’ cite the example of climate activist Greta Thunberg and argue that ‘Thunberg’s efforts have been effective because of her moral reasoning about connections between acts and consequences and the articulation of her position’ (1216; also, see, Bloom, 2010). Again, the thought is that these relationships support moral reasoning as a direct cause of moral progress because moral reasoning is a plausible causal mediator. While Kumar and Campbell’s account will again serve as our main case study, we will also discuss the implications of our criticism for other accounts.

### 3.1. The first step

We have argued that the specific line consists of two steps. First, establish a causal relationship between a predictor that is not moral reasoning and progressive moral beliefs changes; second, argue that moral reasoning can explain that relationship. In what follows, we will present serious challenges to both steps. The next three subsections focus on the first step; we will then turn to the second step.

The aim of the first step is to infer a causal relationship. The gold standard for causal inference in social science are controlled experiments. Since the specific line is based on historical case studies of moral progress, however, it’s clear that meeting this standard is impossible. Instead, the best we can hope for is to infer causality from suitable observational data.

Causal inference based on observational data is hard (e.g. Antonakis, Bendahan, Jacquart, & Lalive, 2010; Hernán & Robins, 2023). Here, we will not go into any technical details; instead, we will focus on three basic requirements. To establish a causal relationship between an outcome Y and a candidate cause X, we need to show that (see, Antonakis et al., 2010, 1087):

1. X correlates with Y.
2. X precedes Y.
3. The relationship between X and Y is not due to any underlying shared causes.

#### 3.1.1. X correlates with Y

Let us begin with the first condition. One point to make here is that Kumar and Campbell provide little direct evidence that people’s factual beliefs improved. Instead, their focus is on establishing that people’s *access* to the relevant facts improved. This makes sense – without systematic surveys, it's difficult to know people's beliefs. However, better access to information does not imply that more people will acquire the information. Consider, for example, that in the a past three decades, the internet has vastly increased people’s access to scientific information, yet levels of average scientific knowledge have barely moved (Besley & Hill, 2020, 23; European Commission, 2021, 19–24). Therefore, even though we think Kumar and Campbell do a reasonable job of establishing that during the time periods they focus on, both the number of people with access to accurate information about the world and the people in it and the proportion of people with progressive moral beliefs increased, this is not quite the correlation they need.

For the sake of argument, let’s grant Kumar and Campbell that during the time periods they focus on, both the proportion of people with accurate beliefs about the world and the people in it and the proportion of people with progressive moral beliefs increased. Notice that this is a population-level claim – the claimed relationship is between two variables that have been aggregated across the entire population. However, recall that the current goal is to establish a (causal) relationship that can be explained by moral reasoning. Since moral reasoning is an individual-level mechanism, to achieve this goal, a population-level correlation is rather unhelpful. Instead, we need an individual-level relationship: factual belief improvements and moral belief improvements need to have happened (often enough) for the same individuals.

But doesn’t population-level correlation imply individual-level correlation? It does not. To see this, consider two sets of people. The first contains people whose factual beliefs improved (in the right way) over the period of time we’re interested in; the second set contains people whose moral beliefs improved in that same time period. We’d only expect to see both a group-level and an individual-level correlation between improved factual and moral beliefs if these sets share a large enough overlap. In contrast, if the two sets do not overlap or if their overlap is small (enough), then we’d only expect to see a population-level correlation, but no individual-level correlation.

We are not saying that this is what happened – we don’t know. Instead, our point is that when pursuing the specific line, researchers need to pay attention to the distinction between population-level and individual-level correlation. To argue that moral reasoning can explain the relationship between access to better factual information (or exposure to public moral speech) and progressive moral belief change, this relationship needs to be established at the level of individuals – this is where moral reasoning does its work. But this is not what Kumar and Campbell or other proponents of the specific line do.

#### 3.1.2. X precedes Y

Suppose we grant that Kumar and Campbell’s case studies reveal an individual-level relationship between factual and moral beliefs. To show this relationship is causal, the time order of the two improvements has to have been right: factual beliefs improve first; progressive moral belief change comes second. As far as we can see, Kumar and Campbell do not address this condition anywhere in their work; neither do any of the other authors who have relied on the specific line. This is a serious problem because of *reverse causality*. In causal inference, especially when human behavior is involved, very often, both ‘X causes Y’ and ‘Y causes X’ are plausible, and so neither can be ruled out a priori (see, Antonakis et al., 2010, 1094). The so-called contact hypothesis provides a useful illustration. According to the contact hypothesis, interactions between members of different groups reduces intergroup prejudice. This is relevant to Kumar and Campbell’s model: in a number of their case studies (2022, 204–205, 208–209, 212–213, 215–216, 220, 231–233), increased inter-group contact is supposed to be a major source of relevant social facts which are the inputs to CR. One common criticism of this hypothesis is that the order of causation may be reversed: the reason we see a negative correlation between inter-group contact and inter-group prejudice may be that when people’s moral attitudes and beliefs about members of the outgroup change, they become less hesitant or even start to seek out contact with outgroup members (see, Pettigrew & Tropp, 2006, 753).

Likewise, reverse causality is an important worry for the proposed causal relationship from public moral speech to progressive moral beliefs. Of course, it is possible that in the historical periods under consideration, public moral speech usually preceded progressive moral belief change. But this need not be so. For example, it may be that many, perhaps most instances of public speech defending or spreading new, moral ideas are delivered by speakers who wanted to jump on the morally progressive bandwagon already well on its way due to other causes.

Again, our point is not to argue for one time sequence over the other. Instead, our aim is to highlight an important detail that moral progress theorists have so far overlooked. When they pursue the specific line, these authors all go into the business of inferring causality from observational data. However, for inferences like that to be convincing, it’s necessary to show that the variables involved have the right time order – otherwise, there is simply no telling whether whatever relationship there may be between them is causal or not, and in what direction the arrow of causality flows.

#### 3.1.3. The threat from confounders

Suppose you want to know if people who keep their cell phone in a breast pocket are at greater risk of lung cancer. You go out and collect data, and find that rates of lung cancer among people who keep their phone in a breast pocket are indeed higher than rates of lung cancer among people who keep their phone elsewhere. Does this mean that cell phone radiation causes lung cancer? No. While out data collecting, you also notice that a lot of people who carry their cell phone in their breast pocket do so because their other pockets are already occupied, including with cigarettes and a lighter. You conclude that it’s unclear if the correlation between cell phone location and lung cancer exists because one causes the other, or because both are caused by a third variable, people’s smoking behavior.

In this example, the causal inference is blocked because of the presence of a confounder – a common cause of both the original candidate cause (cell phone radiation) and the outcome (lung cancer). Confounding is often viewed as the most formidable challenge to inferring causality from observational data, and with good reason: when there are unmeasured or uncontrolled for confounders, it is *never* appropriate to interpret a correlation in causal terms (Antonakis et al., 2010, 1090–1094; Hernán & Robins, 2023, 85–89).

Kumar and Campbell neither mention nor discuss this challenge anywhere in their work; neither do any of the other authors who have used the specific line, as far as we are aware. Yet there clearly are many potential confounders of the relationship between factual beliefs and progressive moral beliefs. One class of potential confounders are individual differences. For example, certain personality traits may make people both more likely to seek out and learn new information about other groups and their members, and to adopt progressive moral beliefs. Religiosity, age, and level of education are other potential examples. Material circumstances may also play the role of confounder. For example, when economic conditions improve, this may cause people to worry less about themselves, leading them to both care more about the moral interests of other groups and to be more interested in learning about these groups and their situation. A third class of potential confounders are to be found in people’s social environment. Social pressure to conform, for example, may cause people to both adopt progressive moral beliefs and relevant factual beliefs about the world and the people in it. Other possible candidates from this domain are status-biased social learning and moral education.

None of these potential confounders strike us as far-fetched. Personality traits like Openness and Agreeableness, for instance, correlate with both people’s information seeking behavior (e.g. Zhang, Yao, Yuan, Deng, & Guo, 2021) and their moral beliefs and values (e.g. Alper & Yilmaz, 2019). Similar correlations have been reported for other individual differences (for information behavior, see, Case, Owen, & Given, 2016; for moral beliefs, see, Graham, Meindl, Beall, Johnson, & Zhang, 2016). Social conformity can have a strong influence on both factual (for a review, see, Cialdini & Goldstein, 2004) and moral beliefs (for a review, see, Chituc & Sinnott-Armstrong, 2020). Finally, socio-economic status is on the one hand a major predictor of many of the moral belief changes moral progress theorists have been interested in (see, Inglehart, 2018); on the other hand, it correlates with information behavior (see, Case et al., 2016) and knowledge about various domains, including social and political issues (for a meta-analysis, see, Hwang & Jeong, 2009).

We are not claiming that any of these specific confounders definitely played a role. Instead, once again, we are simply pointing out an important condition that moral progress researchers pursuing the specific line have so far failed to pay attention to. Even if we knew for sure that in certain historical episodes, factual beliefs or exposure to public moral speech correlated with progressive moral beliefs, this would tell us nothing about the presence or absence of a causal relationship between these variables unless we had good reason to think that there are no confounders blocking the causal inference. But, as we have seen, there are many plausible potential confounders, and so this threat needs to be taken seriously.

### 3.2. The second step

Recall that the specific line involves two steps. First, establish a causal relationship between a predictor (improved relevant factual beliefs or an increase in exposure to relevant public moral speech) and progressive moral beliefs changes; and second, argue that moral reasoning explains that relationship. So far, we have presented a series of challenges to the first step. We will now turn to the second step.

Suppose we’ve managed to identify a historical case where improvements in people’s factual beliefs (or exposure to public moral speech) caused group-level progressive moral belief change. By itself, this would not implicate CR (or any other type of moral reasoning) as a direct cause of moral progress. Instead, CR needs to be the mechanism that underlies the causal relationship.

In social science, the main way to tackle a question like this is causal mediation analysis (Hernán & Robins, 2023, chap. 23). To run an analysis like this, however, one needs to have measured the proposed mediator – clearly, these data are not available in the case of moral reasoning (consistency, or otherwise). It is therefore not surprising that Kumar and Campbell focus their efforts on another, weaker claim: CR provides the best explanation of the relationship between more accurate factual beliefs and progressive moral beliefs.

So, does it? We are not so sure. CR is supposed to be a distinctive type of moral reasoning. Campbell and Kumar (2012) take pains to distinguish CR from other types, including reasoning from general moral principles (291), reasoning by analogy (297) and reflective equilibrium (305). Yet all moral reasoning involves and relies on both moral and non-moral beliefs (see, Harman, Manson, & Sinnott-Armstrong, 2010), and so will often be improved by more accurate factual information. The same is true for domain-general reasoning about moral issues. A causal relationship between improved factual beliefs and progressive moral beliefs, then, wouldn’t be clear-cut evidence for CR – at best, it would be highly ambiguous evidence which doesn't decide between any number of candidate types of reasoning, some moral, others domain-general.

It is not just other types of moral reasoning, though, that could plausibly explain the causal relationship CR is supposed to explain. Another candidate that deserves mention here are affective processes. Learning that members of another group suffer harm, for example, may make people sympathise with their plight, feel guilt or shame about their own role in their treatment, or be outraged at the injustice of it all. Kumar and Campbell at one point seem to suggest these and other affective reactions cannot bring about substantial or lasting moral belief change (2022, 220). However, in support, they only provide one citation (Campbell & Kumar, 2013), which does not report any empirical data or engage with any empirical research. Meanwhile, even though not undisputed, a large body of evidence does implicate various affective processes in the formation of moral judgments and beliefs (for an overview, see, Avramova & Inbar, 2013).

CR, then, clearly is not the only possible explanation of a causal relationship between more accurate factual beliefs and progressive moral beliefs. Is it the most plausible one? Compared to other types of moral reasoning, Kumar and Campbell seem to think so. They deny, for example, that progress on the issue of animal rights can be explained by principle reasoning: ‘ethical treatment of animals is not typically motivated by principles’ (2022, 123). However, Kumar and Campbell provide no evidence or theoretical grounds for this claim. In light of this, CR strikes us as about as likely (or unlikely) an explanation for a relationship between more accurate factual beliefs and progressive moral beliefs as any other form of moral reasoning.

The comparison between CR and affective processes isn’t any more convincing. Again, Kumar and Campbell fail to provide any empirical or theoretical arguments to show that CR is the more likely mediator. Indeed, there is some reason (though not definitive, of course) to think that the opposite is true. Recall that in a number of Kumar and Campbell’s case studies, increased inter-group contact is treated as a major source of relevant social knowledge. A large body of evidence does indeed support a relationship between increased intergroup contact and reduced intergroup hostility and prejudice (e.g. Christ & Kauff, 2019, 146–148; Pettigrew & Tropp, 2006, 757–760). However, when psychologists have investigated the processes which might mediate this relationship, they’ve discovered that intergroup contact seems to leads to improved moral beliefs ‘mainly by reducing negative affect (e.g. intergroup anxiety) and by inducing positive affective processes (e.g. empathy and perspective taking)’, while ‘[c]ognitive mediators (e.g. intergroup knowledge) seem to play a less important role’ (Christ & Kauff, 2019, 152).

The other main version of the specific line faces similar obstacles. Persuasion research offers a number of viable alternative explanations for why people might change their moral beliefs in response to public moral speech, including social expectations and (again) emotional processes (for a review, see, Wood, 2000). Moreover, there is some evidence that the power of moral arguments to change people’s moral beliefs is quite limited (e.g. Stanley, Dougherty, Yang, Henne, & Brigard, 2018), and that when these arguments are effective, this is not primarily because they cause their targets to engage in moral reasoning (e.g. Schwitzgebel, McVey, & May, 2024). Therefore, even if we’d manage to show a causal relationship between exposure to public moral speech and progressive moral belief changes for some historical periods, it is far from obvious that moral reasoning would provide the best (let alone the only) explanation for this relationship.

## 4. Discussion

What drives moral progress is an empirical question, and so any serious attempt to answer will need to be heavily informed by empirical research.^4^ To argue that moral reasoning is a direct cause of moral progress, philosophers and social scientists have presented two lines of argument: the general and the specific line. The general line presents evidence that moral reasoning is in general a powerful mechanism of moral belief change, while the specific line tries to establish that moral reasoning can explain some specific historical examples of moral progress. In this paper, we have provided a detailed breakdown of both lines, using Kumar and Campbell’s (2022) model of rational moral progress as a case study. We explained the empirical assumptions made in each line and argued that the available evidence does not support any of these assumptions convincingly.

Our main point is not that any of these accounts are incorrect – we don’t know. Instead, it is that the evidence these authors provide is so unconvincing and insufficient that it does not (and, in our opinion, should not) move our priors at all.

You may protest that we are being unfair because the goal of these authors isn’t to make a convincing empirical case for moral reasoning as a direct cause of moral progress, but is better understood as theory crafting – coming up with bold ideas for social scientists to test. If so, our paper is still useful because it spells these ideas out in detail and elaborates a list of standards that can help guide those who are interested in assembling and evaluating the evidence for them. However, it is clear that most of the authors whose work we discussed do not take themselves to be (just) theory crafters. Instead, they take it that the evidence they provide establishes their ideas to a point where these ideas can help us predict and even bring about future moral progress. Kumar and Campbell, for example, advertise their model as providing a way to achieve moral progress that is ‘more reliable and durable than moral progress by any other means’ (2022, 196), and give prescriptions (including to activists) for how to achieve future moral progress on the issues of animal welfare, transgender rights, racial inequality, class inequality and climate injustice (2022, 214–250). Many other authors have made similar claims (e.g. Bloom, 2010; Killen & Dahl, 2021, 1219–1221; Kitcher, 2021; Tam, 2020, 92–94; Singer, 2011, 88). If a theory about the causes of moral progress is going to inform prescriptions for how people should act to bring about future moral progress (or avert future moral regress), then this link to practical action brings with it a high evidential requirement for that theory.

You may also protest that we are holding these authors and the evidence they present to standards that are too high. We disagree. These authors are making far-reaching claims about the causes of human thought and behavior in large, extremely complex (that is, involving a huge number of interacting, moving parts) social environments. Claims like this are extremely ambitious, and so making a convincing case for them is simply very challenging. Indeed, this is how it should be if we are going to use such claims as a basis for action and policy.

Say you find our arguments convincing. What does this mean for research on the mechanisms of progressive moral belief change going forward? When it comes to moral reasoning as a direct cause of moral progress, we believe that the specific line is, at least for the moment, dead. The main reason is that the data we would need for robust causal inference simply does not exist for any historical case study that we know of. This includes systematic measurements of the outcome (progressive moral belief changes), direct measurements of people’s engagement in moral reasoning, and measurements of potential confounders (of which there are many, as we have seen) – all preferably within-subject (see, e.g. Hernán & Robins, 2023, chaps. 11–23; Antonakis et al., 2010). Perhaps moral progress researchers of the future at some point may have data like these available to them for progressive episodes that have not yet happened; until this time, we don’t see much to be gained from further pursuing the specific line.

In contrast, we think there are a number of steps that could be taken to shore up the general line. To once more use consistency reasoning as an illustration, instead of relying on findings about moral order effects, researchers could instead devise studies to investigate CR and its effect on moral beliefs more directly. Also, there are ways to design studies that have a better shot at telling us about the role of consistency reasoning in real-life moral cognition, about how often people engage in it, with what effects and whether these effects typically persist over time (cf., Bollich et al., 2016; Hofmann, Wisneski, Brandt, & Skitka, 2014). None of this would not be easy; however, it is possible to do, and the results would allow us a much better sense of the progressive potential of consistency reasoning (or other types of moral reasoning).

## Notes

1 We mean moral beliefs to include related mental states like moral judgments, attitudes, and values.

2 In the accounts we critique in this paper, the ‘thing’ that improves are people’s moral beliefs. We should point out that not all work on moral progress treats moral beliefs as the (main) unit of change and that some moral progress theorists instead focus on improvements in people’s moral reasoning abilities (e.g., Moody-Adams, 1999). However, note that moral reasoning plays a very different role in these accounts compared to the accounts we discuss in this paper: the former view moral reasoning as the ‘thing’ that improves during moral progress, while the latter view moral reasoning as a major cause of moral belief improvement.

3 There isn’t a clear threshold for when a proportional change counts as substantial. That said, many common examples of moral progress in the literature involve populations where the proportion of the population who changed their moral beliefs exceeds or is supposed to have exceeded 50% (e.g., the abolition of slavery; gay rights; equality of men and women; the abolition of cruel and unusual punishments).

4 Note that this research need not come from psychology (or related disciplines). This paper focused on accounts of moral progress that propose moral reasoning as a major direct cause of moral progress. Since moral reasoning is something that happens in individual people’s heads, psychology is the most natural discipline to look to for empirical support. Other theories of what drives moral progress do not invoke psychological causes, but instead focus on social and political institutions (Jaeggi, 2021), social movements (Anderson, 2014) or people’s physical and economic security (Buchanan & Powell, 2018; Inglehart, 2018), for example. Of course, to what extent any of these accounts succeed is also an empirical question; yet to answer it, the most relevant evidence will likely not come from psychology, but from disciplines like history, political science and economics.
